# Burnout: Fifty Years Later

**DOI:** 10.1177/21650799241260441

**Published:** 2024-07-27

**Authors:** Renzo Bianchi, Gail Swingler, Irvin Sam Schonfeld

**Affiliations:** 1 Norwegian University of Science and Technology (NTNU); 2 The City College of the City University of New York

**Keywords:** burnout, prevalence, diagnosis, etiology, job stress, depression, occupational health, stigma, exhaustion, fatigue

Burnout has elicited considerable interest among occupational health specialists since it was first described in the mid-1970s. The publication of the Maslach Burnout Inventory (MBI) in 1981 stimulated research on the syndrome and established *exhaustion, cynicism*, and *inefficacy* as its defining components (Maslach et al., 2016). Health professionals are thought to be particularly affected by burnout, with detrimental effects on absenteeism, presenteeism, job performance and turnover, and general health status (Rotenstein et al., 2018; Woo et al., 2020). Five decades of research on burnout have led stakeholders to revise their views of the phenomenon. This article outlines key developments in burnout research that have unfolded over this period.

First, new light has been shed on burnout’s etiology. Most burnout researchers have assumed that burnout results from intractable job stress (e.g., work overload). Meta-analyses indicate that job stressors indeed predict burnout, but only tenuously (e.g., Guthier et al., 2020). That intractable job stress accounts for little variance in burnout may explain why interventions exclusively centered on the workplace have shown limited effectiveness. Second, although burnout has been portrayed as rampant, researchers have shown that burnout prevalence is unmeasurable for the basic reason that burnout is undiagnosable (e.g., Rotenstein et al., 2018). As underscored by Maslach and Leiter (2017) regarding the MBI: “. . . no clinical research has been done to establish that any particular score, or pattern of scores, is a meaningful indicator of serious problems in well-being or work performance” (p. 38). Third, evidence has accumulated that burnout cannot be easily distinguished from a depressive condition (Bianchi et al., 2021; Sen, 2022). Clinicians have increasingly questioned the once-common recommendation of *not* conflating burnout with depression. The risk of withholding life-saving treatments for depression from individuals categorized as “burned-out” has raised deep concerns (Bianchi & Schonfeld, 2023; Sen, 2022). Fourth, while burnout has been viewed as a socially accepted label, there is mounting evidence that the burnout label is stigmatizing and should be handled carefully in organizational settings (e.g., Smith et al., 2023). Finally, the definition of burnout as a syndrome of exhaustion, cynicism, and inefficacy has been challenged. Investigators have noted that, when describing stressed-out workers, exhaustion, cynicism, and inefficacy only represent bits and pieces of the story—and arguably not the most relevant ones (Bianchi & Schonfeld, 2023). The MBI developers themselves recognized that exhaustion, cynicism, and inefficacy do not constitute “a single, one-dimensional phenomenon” (Maslach et al., 2016, p. 72). Such an avowal is consequential. The notion that exhaustion, cynicism, and inefficacy constitute disparate entities that do not coalesce into a unified phenomenon drains the burnout construct of its substance.

Deep controversies continue to surround burnout, often leaving stakeholders confused. A shift in focus from burnout to job-related forms of anxiety and depressive symptoms may allow occupational health specialists to support workers’ health and well-being more effectively.
